# DYSBIOSIS OF GUT MICROBIOTA IN PATIENTS HOSPITALIZED FOR THE TREATMENT OF ALCOHOL USE DISORDER

**DOI:** 10.1590/S0004-2803.24612025-144

**Published:** 2026-10-26

**Authors:** Rutiane Ullmann THOEN, Larisse LONGO, Bruno de Souza BASSO, Pabulo Henrique RAMPELOTTO, Anne Orgler SORDI, Félix Henrique Paim KESSLER, Lisia Von DIEMEN, Mário Reis ÁLVARES-DA-SILVA

**Affiliations:** 1 Universidade Federal do Rio Grande do Sul, Programa de Pós-Graduação em Gastroenterologia e Hepatologia, Porto Alegre, RS, Brasil.; 2 Hospital de Clínicas de Porto Alegre, Centro de Pesquisa Experimental, Laboratório Experimental de Hepatologia e Gastroenterologia, Porto Alegre, RS, Brasil.; 3 Universidade Federal do Rio Grande do Sul, Programa de Pós-Graduação em Ciências Biológicas: Farmacologia e Terapêutica, Porto Alegre, RS, Brasil.; 4 Universidade Federal do Rio Grande do Sul, Programa de Pós-Graduação em Psiquiatria e Ciências do Comportamento, Porto Alegre, RS, Brasil.; 5 Universidade Federal do Rio Grande do Sul, Hospital de Clínicas de Porto Alegre, Centro de Pesquisas sobre Drogas e Álcool e Centro Colaborador em Álcool e Drogas, Porto Alegre, RS, Brasil.; 6 Hospital de Clínicas de Porto Alegre, Divisão de Gastroenterologia, Porto Alegre, RS, Brasil.

**Keywords:** Alcohol use disorder, alcohol-related liver disease, inflammatory markers, microbiota, Transtorno por uso de álcool, doença hepática relacionada ao álcool, marcadores inflamatórios, microbiota intestinal

## Abstract

**Background and objectives:**

Alcohol Use Disorder (AUD) is a global issue with a significant financial impact on health. The main objective of this study was to assess the chronic effect of alcohol on the composition and diversity of intestinal microbiota in AUD patients.

**Methods:**

Prospective cohort study in patients hospitalized for AUD treatment. Demographic, clinical, and laboratory data and inflammatory markers were assessed at the time of hospital admission for AUD-T1-group and in the control group, as well evaluation of the gut microbiota composition, while the AUD-T2-group was assessed at the discharge from hospital treatment.

**Results:**

50 participants were included in the control group and 47 admitted for AUD treatment. The mean age in the AUD-T1-group was 49.3 years, all male. Mieloperoxidase concentration was significantly higher in AUD-T1-group compared to control (*P*<0.001). The opposite was observed for E-selectin (*P*<0.001). Global alpha diversity was different among the control, AUD-T1 and AUD-T2 groups, ACE index (*P*<0.004), Shannon index (*P*<0.017), observed taxa (*P*=0.002). The LEfSe algorithm identified a higher abundance of *Faecalibacterium*, *Bacteroides*, *Agathobacter*, and *Ruminococcaceae unclassified* in control compared to the AUD-T1. There were no differences in the abundance of bacterial genera between the AUD-T1 and AUD-T2 groups.

**Conclusion:**

Patients with AUD exhibit changes in intestinal microbiota composition, potentially associated with low-grade inflammation and functional alterations that contribute to the development of diseases. The composition of intestinal microbiota and its bioactive metabolites may serve as novel therapeutic targets for the treatment of AUD.

## HIGHLIGHTS

• Chronic alcohol consumption is associated with gut microbiota dysbiosis.

• Alcohol use disorder patients show distinct inflammatory marker profiles compared to healthy controls.

• Reduced abundance of key commensal bacteria may contribute to alcohol-related disease mechanisms.

• Gut microbiota abnormalities persist despite short-term alcohol withdrawal.

## RESUMO

**Contexto e objetivos:**

O Transtorno por Uso de Álcool (TUA) é um problema global com impacto financeiro significativo para os sistemas de saúde. O principal objetivo deste estudo foi avaliar o efeito crônico do álcool sobre a composição e a diversidade da microbiota intestinal em pacientes com TUA.

**Métodos:**

Estudo de coorte prospectivo realizado com pacientes hospitalizados para tratamento do TUA. Foram avaliados dados demográficos, clínicos e laboratoriais, bem como marcadores inflamatórios no momento da admissão hospitalar (grupo TUA-T1) e no grupo controle. Também foi analisada a composição da microbiota intestinal, enquanto o grupo TUA-T2 foi avaliado na alta hospitalar após o tratamento.

**Resultados:**

Foram incluídos 50 participantes no grupo controle e 47 pacientes internados para tratamento do TUA. A idade média no grupo TUA-T1 foi de 49,3 anos, sendo todos do sexo masculino. A concentração de mieloperoxidase foi significativamente maior no grupo TUA-T1 em comparação ao grupo controle (*P*<0,001). O oposto foi observado para a E-selectina (*P*<0,001). A diversidade alfa global diferiu entre os grupos controle, TUA-T1 e TUA-T2, conforme demonstrado pelo índice ACE (*P*<0,004), índice de Shannon (*P*<0,017) e número de táxons observados (*P*=0,002). O algoritmo LEfSe identificou maior abundância de *Faecalibacterium*, *Bacteroides*, *Agathobacter* e *Ruminococcaceae* não classificadas no grupo controle em comparação ao grupo TUA-T1. Não foram observadas diferenças na abundância dos gêneros bacterianos entre os grupos TUA-T1 e TUA-T2.

**Conclusão:**

Pacientes com TUA apresentam alterações na composição da microbiota intestinal, potencialmente associadas à inflamação de baixo grau e a alterações funcionais que contribuem para o desenvolvimento de doenças. A composição da microbiota intestinal e seus metabólitos bioativos podem representar novos alvos terapêuticos para o tratamento do TUA.

## INTRODUCTION

Alcohol use disorder (AUD) represents a global health problem, and the burden of excessive alcohol consumption has increased over time^1^ ^,^ ^2^. According to the World Health Organization (WHO), approximately 2.3 billion people are current consumers of alcohol, and around 1 billion of them are considered heavy episodic drinkers^3^ ^,^ ^4^. Chronic alcohol consumption is related to more than 200 disease and injury outcomes, generating burdens for health, society, and economy^1^ ^,^ ^3^. In patients with AUD, alcoholic liver disease (ALD) should be evaluated as it can lead to extrahepatic manifestations that affect various systems, such as the nervous and gastrointestinal^2^ ^,^ ^3^ ^,^ ^5^. Liver cirrhosis is one of the leading causes of death in adults, with alcohol abuse accounting for a significant proportion, representing 48% of all related deaths^2^ ^,^ ^5^. In clinical practice, it is crucial to assess characteristics associated with alcohol consumption, such as type of drink, duration, and pattern of consumption, as these factors are usually connected to the patient’s clinical condition. However, quantifying alcohol consumption in daily practice is not simple^3^ ^,^ ^5^ ^,^ ^6^. The European Association for the Study of the Liver and the WHO have recommended standardizing a “drink” as 10 grams of pure alcohol^7^. However, depending on the geographic location, there is a divergence in the amount of grams of alcohol per dose, for example: 10 g in Europe, 14 g in the US and Latin America, and 23 g in Japan^8^. Despite that, heavy episodic drinking is defined as consuming more than 60 grams of pure alcohol on a single occasion at least once a month, while binge drinking is characterized by consuming four or more drinks for women or five or more drinks for men within a short period of time^8^.

It is known that there is a strong correlation between the quantity and duration of alcohol consumption and the progression of liver disease^3^ ^,^ ^5^ ^,^ ^8^. It is postulated that genetic and epigenetic factors, metabolic dysfunction, and alterations in the composition of the gut microbiota also play a crucial role in the development and progression of liver damage^3^ ^,^ ^5^ ^,^ ^8^. In summary, dysfunction of the intestine-liver axis, induced by excessive and chronic alcohol consumption, leads to changes in the composition of the gut microbiota and increased intestinal permeability^2^ ^,^ ^3^ ^,^ ^6^. These factors stimulate bacterial translocation, the release of lipopolysaccharides, and endotoxemia, contributing to the inflammatory processes and fibrogenesis^2^ ^,^ ^3^ ^,^ ^6^. However, there is still limited understanding of how the composition of the gut microbiota responds when patients diagnosed with AUD cease drinking, which is an objective of this study. Alcohol-associated memories and cravings play a crucial role in the development and maintenance of AUD^9^ ^,^ ^10^.

Currently available pharmacotherapeutic options have limited efficacy in the treatment of AUD, and new treatment strategies are focused on manipulating memories associated with alcohol due to the dysregulation of the neuroimmunological system^9^ ^-^ ^12^. Therefore, this study was developed with the main objective of evaluating the effect of chronic alcohol intake on the composition and diversity of the gut microbiota in patients with AUD.

## METHODS

Prospective cohort study carried out in patients hospitalized due to excessive and chronic alcohol consumption at a university hospital in southern Brazil. To carry out this study, male individuals aged over 18 years were included. Participants were required to meet the Diagnostic and Statistical Manual of Mental Diseases (DSM)-IV criteria AUD upon admission to the study. Patients with human immunodeficiency virus (HIV) infection, coronavirus disease-19, suspected or diagnosed metabolic dysfunction-associated steatotic liver disease, transplant recipients, patients with other chronic inflammatory diseases such as rheumatological and inflammatory bowel diseases, were excluded. Additionally, patients using immunosuppressants and those unable to provide the necessary biological samples for the study were excluded. To integrate the control group (CON), participants of both genders, over 18 years old, who did not have a diagnosis of chronic disease, denied the use of prescribed medications and use of antibiotics in the six months prior to inclusion in the study, were recruited.

The patients admitted for AUD treatment had their clinical, laboratory, and biological samples collected upon hospital admission (AUD-T1). On the 14th day of hospitalization (AUD-T2), the date when research participants were discharged, only feces collection was performed. Data and biological samples from the CON group were collected at a single time point. This study was approved by the Ethics Committee of Hospital de Clínicas de Porto Alegre (CAAE 24130519.3.0000.5327 and CAAE 26760119.0.0000.5327) and follows the guidelines for studies in humans.

### Demographic, clinical and laboratory variables

At the time of hospital admission for AUD treatment (AUD-T1), demographic, laboratory, and clinical data were collected directly from the patients’ medical records. Measurements of waist circumference, height, and weight were taken, and body mass index (BMI) was calculated. Additionally, on the first day of hospitalization, a blood sample was collected from each patient to detect serum alterations related to markers of endothelial and vascular dysfunction. Through the multiplex assay with the Luminex platform (Millipore, Germany) we performed the evaluation of intercellular adhesion molecules-1 (ICAM-1), vascular cell adhesion molecules (VCAM-1), E-selectin and myeloperoxidase (MPO). The results were expressed in ng/ml. All procedures were performed according to the manufacturer’s instructions, and the analysis was conducted in duplicate. This assessment was carried out on patients in the AUD-T1 and control groups.

### DNA extraction and 16S rRNA sequencing

The participants received instructions on the proper procedures for collecting fecal samples. The collection was conducted on the day of hospital admission (AUD-T1) and on the 14th day (AUD-T2). The analysis of the composition and diversity of the gut microbiota was performed on the fecal samples obtained from the participants hospitalized for AUD treatment (AUD-T1 and AUD-T2) as well as the control group.

The bacterial DNA was isolated from the fecal samples using the QIAamp fast DNA stool mini kit (Qiagen, USA), following the manufacturer’s instructions. The hypervariable V4 region from the rRNA gene was amplified by PCR using the following primer pair: 515F (5’-GTGCCAGCMGCCGCGGTAA-3’) and 806R (5’-GGACTACHVGGGTWTCTAAT-3’). To pool different samples in the same reaction, we used the primer-fusion method and each sample had a distinct barcode attached on the corresponding PCR product. The purified products were subjected to emulsion PCR using Ion PGM™ Hi-Q™ view OT2 kit (Thermo Fisher Scientific, USA). After, the resulting enriched beads were sequenced in a next-generation sequencing machine using Ion PGM™ Hi-Q™ view sequencing kit (Thermo Fisher Scientific, USA).

### Bioinformatics analyses

The sequence data exported from the Ion PGM™ System was processed using a custom pipeline in Mothur v.1.41.1^13^. Initially, sequences were depleted of barcodes and primers (where no mismatch was allowed) and then a quality filter was applied to eliminate low quality reads. Quality control was conducted by trimming the low-quality reads, those with incorrect length, those containing an ambiguous base, or containing homopolymers longer than 8bp. All potentially chimeric sequences were identified and removed using VSEARCH^14^. After this initial quality, filtering and trimming steps, the remaining sequences were clustered into operational taxonomic units (OTUs) based on a 99% identity level and were classified against the SILVA v132 reference database at 97% similarity^15^. Sequences that could not be classified (i.e., “unknown” sequences), as well as sequences identified as eukaryotes, mitochondria, and chloroplasts were removed prior to further analysis. The resulting OTU table was rarefied to the smallest library size. Subsequent analyses of the sequence dataset were performed in R v. 4.0.0 (using vegan, phyloseq, ggplot2, and MicrobiomeAnalystR packages).

Alpha diversity was assessed using the number of observed taxa as well as ACE and Shannon index. For the overall comparison of significant differences among bacterial communities (i.e., beta diversity), principal coordinates analysis (PCoA) was performed. A matrix using the Bray-Curtis dissimilarity metric was calculated for each pair of samples. To achieve statistical confidence for the sample grouping observed by PCoA, the ANOSIM multivariate test was performed on the distance matrix. To compare additional differences among the microbial communities, clustering methods based on Bray-Curtis dissimilarity were performed. The results of hierarchical clustering were visualized using dendrograms. To identify differentially abundant taxa at the phylum, family, and genus level, the linear discriminant effect size (LEfSe) method was performed. The algorithm performs a nonparametric factorial Kruskal-Wallis sum rank test and linear discriminant analysis (LDA) to determine statistically significant different features among taxa and estimates the effect size of the difference. Differences were considered significant for a logarithmic LDA score threshold of ±1.5 and a *P*<0.05.

### Sample size and statistical analysis

The sample size was determined using the online version of the Power and Sample Size for Health Researchers tool, based on the study published by Sacanella et al., which demonstrated alterations in serum levels of endothelial and vascular markers in individuals with chronic alcoholism^16^. Considering a power of 80% and a significance level of 5%, it was determined that 44 participants needed to be recruited to form the group of patients hospitalized for AUD.

Data symmetry was tested using the Shapiro-Wilk test. Quantitative variables were expressed as mean ± standard deviation or median interquartile range (25th-75th). Categorical variables were expressed as frequencies and percentages. For symmetrical variables, the independent t-test was used, and asymmetric data were evaluated using the Mann-Whitney U test. The chi-square test was used to test the significance of the association between variables. *P*<0.05 was considered statistically significant. Data were analyzed in the Statistical Package for Social Sciences 21.0 (SPSS Inc., USA).

## RESULTS

For the development of the study, a total of 47 patients who were hospitalized for AUD treatment were assessed. Among them, 44 individuals (93.6%) fulfilled the inclusion criteria and were included in the follow-up (SUPPLEMENTARY FIGURE 1). Additionally, 50 participants were recruited into the CON group, with no screening failures.

**SUPPLEMENTARY FIGURE:**
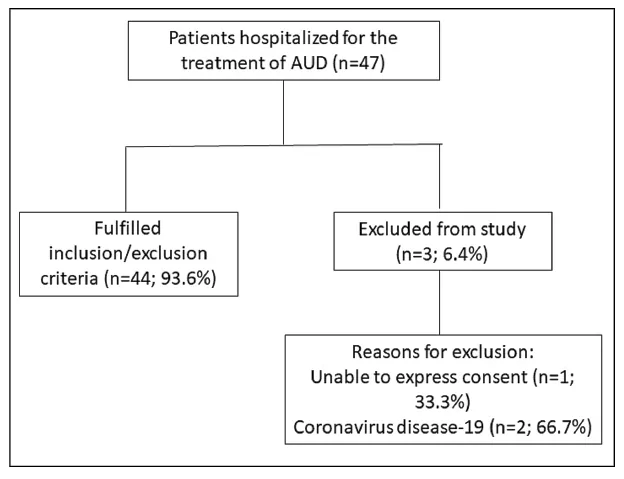
1. Consort diagram for the group of patients hospitalized for the treatment of Alcohol use disorder (AUD).

Demographic, clinical and biochemical characteristics of patients hospitalized for the treatment of alcohol use disorder

The demographic and clinical data in patients hospitalized for the treatment of AUD are shown in Table 1. The average age of participants diagnosed with AUD-T1 was 49.3 (±8.9) years, all of whom were male. Abdominal circumference was significantly higher in patients with AUD-T1 compared to the control group (*P*<0.001), however, no significant difference was observed between the groups for BMI (*P*=0.073). At the time of admission for AUD-T1, 21 (47.7%) participants were active smokers, 6 (13.6%) had a diagnosis of diabetes mellitus, and 5 (11.4%) had hepatic cirrhosis. Cases of hepatic cirrhosis are associated with excessive alcohol consumption, and in two situations, there is an association with hepatitis C virus infection.

**TABLE 1:** Demographic and clinical characteristics of patients hospitalized for the treatment Of AUD compared to the control group.

Variables*	Control (n=50)	AUD-T1 (n=44)	***P*#**
Age (years)	41.2 ± 17.1	49.3 ± 8.9	0.006
Male gender	12 (24.0%)	44 (100%)	<0.001
Waist circumference (cm)	86.6 ± 9.9	97.7 ± 13.3	<0.001
BMI (kg/m^2^)	24.4 ± 3.1	25.8 ± 5.3	0.073
Active smoke	0 (0.0)	21 (47.7%)	-
Diabetes *mellitus*	0 (0.0)	6 (13.6%)	-
Cirrhosis	0 (0.0)	5 (11.4 %)	-

*Variables expressed as frequency (%) and mean ± standard deviation. *# P*<0.05 was considered statistically significant. No statistical analysis was performed between the groups for the variables active smoking, diabetes *mellitus*, and cirrhosis, since no participant in the control group had these clinical conditions. AUD: alcohol use disorder. BMI: body mass index.

The results regarding the laboratory parameters at the time of hospital admission for patients with AUD-T1 are reported in Table 2. According to the reference values used in the institution, AUD-T1 patients had elevated serum levels of alanine aminotransferase (ALT), aspartate aminotransferase (AST), gamma-glutamyl transferase (GGT), and C-reactive protein (CRP). No biochemical and hematological changes were observed for the other evaluated parameters in this population at the time of hospital admission.

**TABLE 2:** Laboratory data at the time of hospital admission of patients with AUD.

Variables*	AUD-T1 (n=44)	Reference values
Glucose (mg/dL)	95.3 ± 28.8	<100
Triglyceride (mg/dL)	126.1 ± 63.0	<150
Total cholesterol (mg/dL)	189.5 ± 40.2	<190
HDL cholesterol (mg/dL)	57.8 ± 26.6	>40
LDL cholesterol (mg/dL)	106.7 ± 28.9	<100
ALT (U/L)	55.8 ± 61.5	<55
AST/ALT	66.3 ± 84.8	5 - 34
GGT (U/L)	173.3 ± 276.6	<55
Sodium (mEq/L)	139.0 ± 3.2	136 - 145
Potassium (mEq/L)	4.3 ± 0.5	3.5 - 5.1
Magnesium (mEq/L)	1.9 ± 0.2	1.6 - 2.6
Albumin (g/dl)	3.9 ± 0.5	3.5 - 5
Creatinine (mg/dL)	0.8 ± 0.2	0.72 - 1.25
Urea (mg/dL)	26.1 ± 12.1	18 - 55
Total bilirubin (mg/dL)	0.6 ± 0.3	0.2 - 1.2
Indirect bilirubin (mg/dL)	0.4 ± 0.2	<0.7
CRP (mg/L)	47.2 ± 35.6	≤5
Lipase (U/L)	40.9 ± 31.0	<60
TP (%)	91.2 ± 17.0	>70
Platelets (x10^3^/µl)	225.6 ± 74.6	150 - 400

Variables described as mean ± standard deviation. AUD: alcohol use disorder. ALT: alanine aminotransferase. AST: aspartate aminotransferase. GGT: gamma-glutamyl transferase. HDL: high-density lipoprotein. LDL: low-density lipoprotein. CRP: C-reactive protein. TP: prothrombin time.

### Markers of endothelial and vascular dysfunction

The results obtained for the markers of endothelial and vascular dysfunction are shown in Figure 2. There was a significant increase in the serum concentration of MPO among the participants hospitalized for AUD-T1 treatment compared to the control group (*P*<0.001). Conversely, the concentration of e-selectin was significantly (*P*<0.001) lower in the AUD-T1 group compared to the control participants. There were no significant differences observed between the groups in terms of VCAM-1 (*P*=0.194) and ICAM-1 (*P*=0.205) concentrations.

**FIGURE 1:**
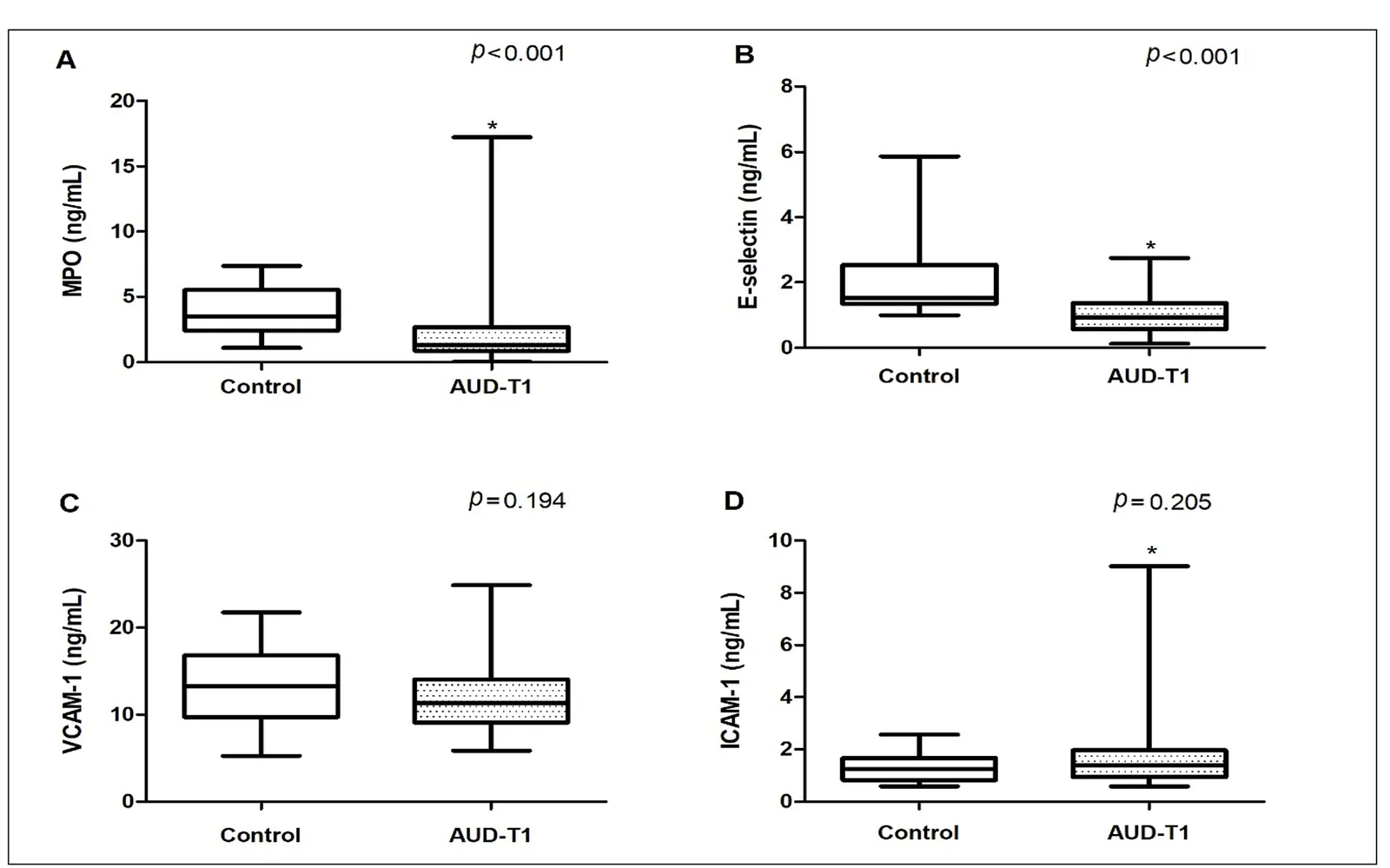
Serum concentration of markers of endothelial and vascular dysfunction. (A) MPO, (B) e-selectin, (C) VCAM-1, and (D) ICAM-1. Data expressed as median (25th-75th percentile), and *P*<0.05 was considered statistically significant. AUD-T1: hospital admission for alcohol use disorder treatment. ICAM-1: intercellular adhesion molecules-1. MPO: myeloperoxidase. VCAM-1: vascular cell adhesion molecules.

### Gut microbiota diversity and composition

In terms of global alpha diversity, significant differences were observed between the study groups in terms of ACE index (*P*<0.004; Figure 2A), Shannon index (*P*<0.017; Figure 2B), and observed taxa (*P*=0.002; Figure 2C). To explore the global differences in gut bacterial communities among groups, a PCoA based on Bray-Curtis distances of OTUs was conducted. However, upon examining the PCoA ordination plot, no significant differences were observed in the structural pattern of the gut microbiota among the groups (ANOSIM *P*=0.936; Figure 2D).

**FIGURE 2:**
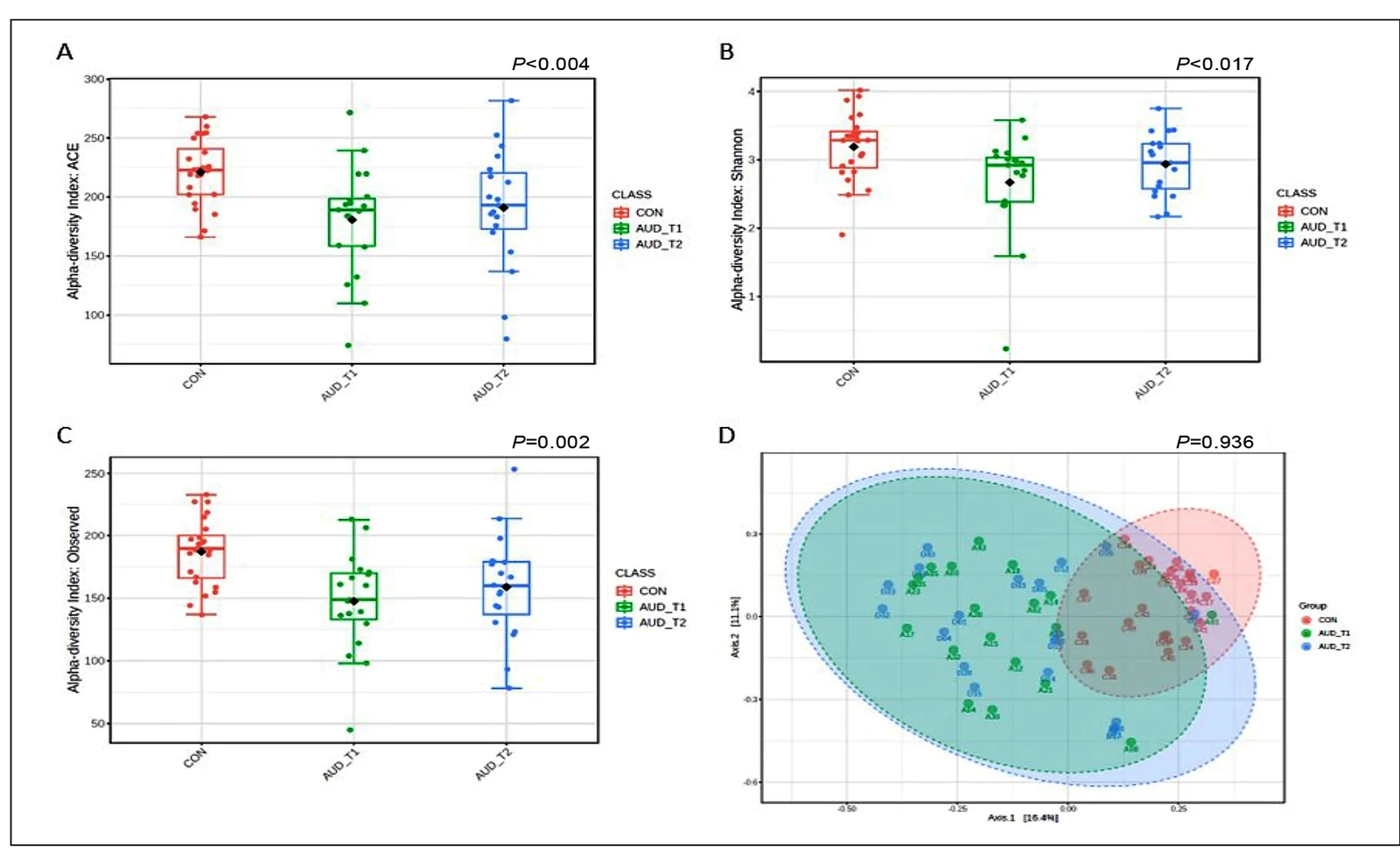
Analysis of global alpha and beta diversity using various metrics. (A) ACE index, (B) Shannon index, (C) Observed taxa and, (D) PCoA based on Bray-Curtis. Statistical confidence was accessed using the multivariate Kruskal-Wallis test. *P*<0.05 was considered statistically significant. AUD-T1: hospital admission for alcohol use disorder treatment. AUD-T2: hospital discharge after alcohol use disorder. CON: control.

Following that, we conducted the statistical analysis of alpha and beta diversity between the AUD-T1 and AUD-T2 groups, aiming to evaluate and compare these variables at the time of hospital admission and discharge for AUD participants in the study. There were no significant differences in terms of alpha diversity concerning the ACE index (*P*=0.234; Figure 3A), Shannon index (*P*=0.246; Figure 3B), and observed taxa (*P*=0.286; Figure 3C). However, significant differences were observed in the structural pattern of the gut microbiota in the PCoA analysis based on Bray-Curtis distances between the AUD-T1 and AUD-T2 groups (ANOSIM *P*<0.001; Figure 3D).

**FIGURE 3:**
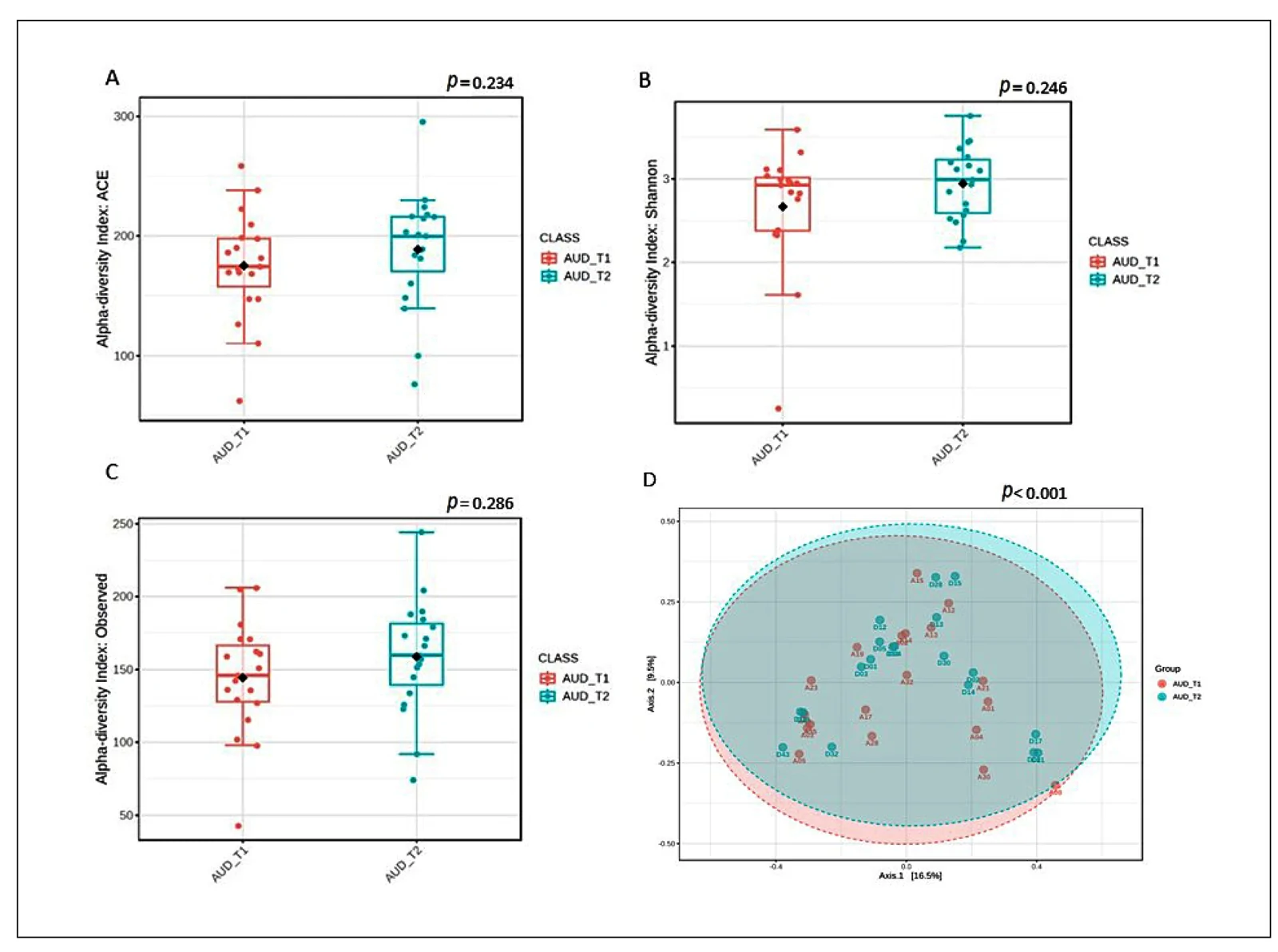
Analysis of alpha and beta diversity between the AUD-T1 and AUD-T2 using various metrics. (A) ACE index, (B) Shannon index, (C) Observed taxa and, (D) PCoA based on Bray-Curtis. Statistical confidence was accessed using the multivariate Kruskal-Wallis test. *P*<0.05 was considered statistically significant. AUD-T1: hospital admission for alcohol use disorder treatment. AUD-T2: hospital discharge after alcohol use disorder. CON: control.

Taxonomy-based analysis of bacterial communities identified 1506 bacterial taxa (OTUs), belonging to 182 genera, 66 families, and 9 phyla. *Firmicutes* (63%), *Bacteroidetes* (26%), and *Proteobacteria* (5%) were the most prevalent phyla in all samples. These three phyla were also differentially abundant among groups. Other phyla had less than 4% abundance each. The most abundant families were *Ruminococcaceae* (22%), *Lachnospiraceae* (18%), *Bacteroidaceae* (12%), and *Prevotellaceae* (12%). These four families represented 64% of all observed taxa. The distribution of taxonomic taxes for the families *Ruminococcaceae*, *Lachnospiraceae*, and *Bacteroidaceae* is more prevalent in the control group, while the presence of *Prevotellaceae* was more prevalent in the AUD-T1 group. The 15 most abundant genera, with more than 2% relative abundance, represented 77% of all observed taxa in this study and may be considered a core microbiome.

To confirm both statistical and biological taxonomic differences at the genus level among groups, the LEfSe algorithm with a logarithmic LDA score cutoff ≥2.0 was used. In total, the differential abundance analysis identified seven differentially abundant genera. Among these identified bacterial genera, *Faecalibacterium*, *Bacteroides*, *Agathobacter*, and *Ruminococcaceae_unclassified* were more abundant in the control group compared to the AUD-T1 and AUD-T2 groups (Figure 4). The opposite was reported for the genera *Prevotella_9*, *Enterobacteriaceae_unclassified*, and *Catenibacterium* (Figure 5). There were no differences in bacterial genus abundance between the AUD-T1 and AUD-T2 groups.

**FIGURE 4:**
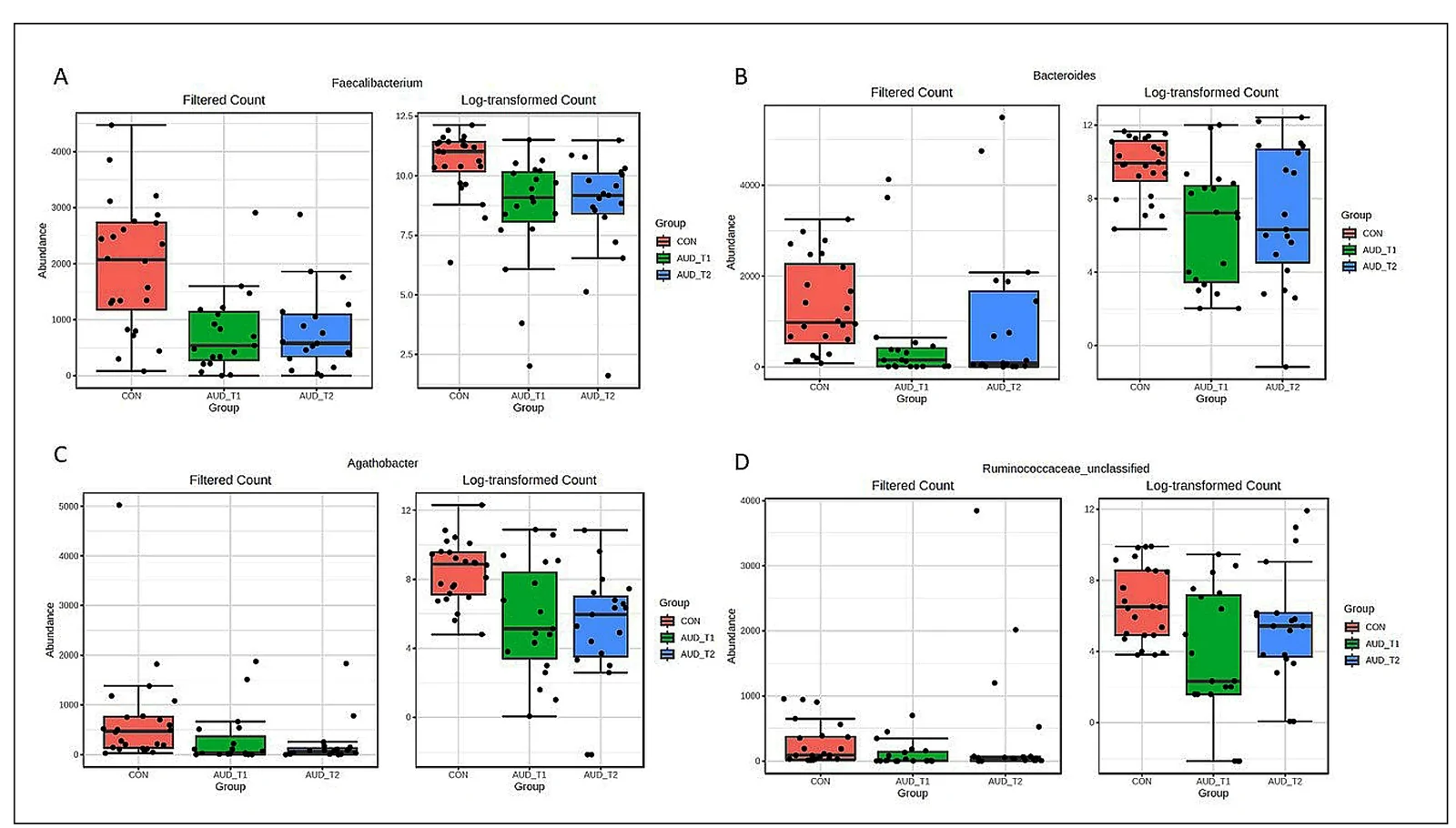
Differential abundant OTUs between the control, AUD-T1, and AUD-T2 groups, by Lefse analysis. (A) genera *Faecalibacterium*, (B) genera *Bacteroides*, (C) genera *Agathobacter*, and, (D) genera *Ruminococcaceae_unclassified*. Taxa were ranked by LDA score. Differences were considered significant for a logarithmic LDA score threshold of ±2.0 and *P*-value <0.05. AUD-T1: hospital admission for alcohol use disorder treatment. AUD-T2: hospital discharge after alcohol use disorder. CON: control. LDA: linear discriminant analysis. Lefse: linear discriminant effect size. OTUs: operational taxonomic units.

**FIGURE 5:**
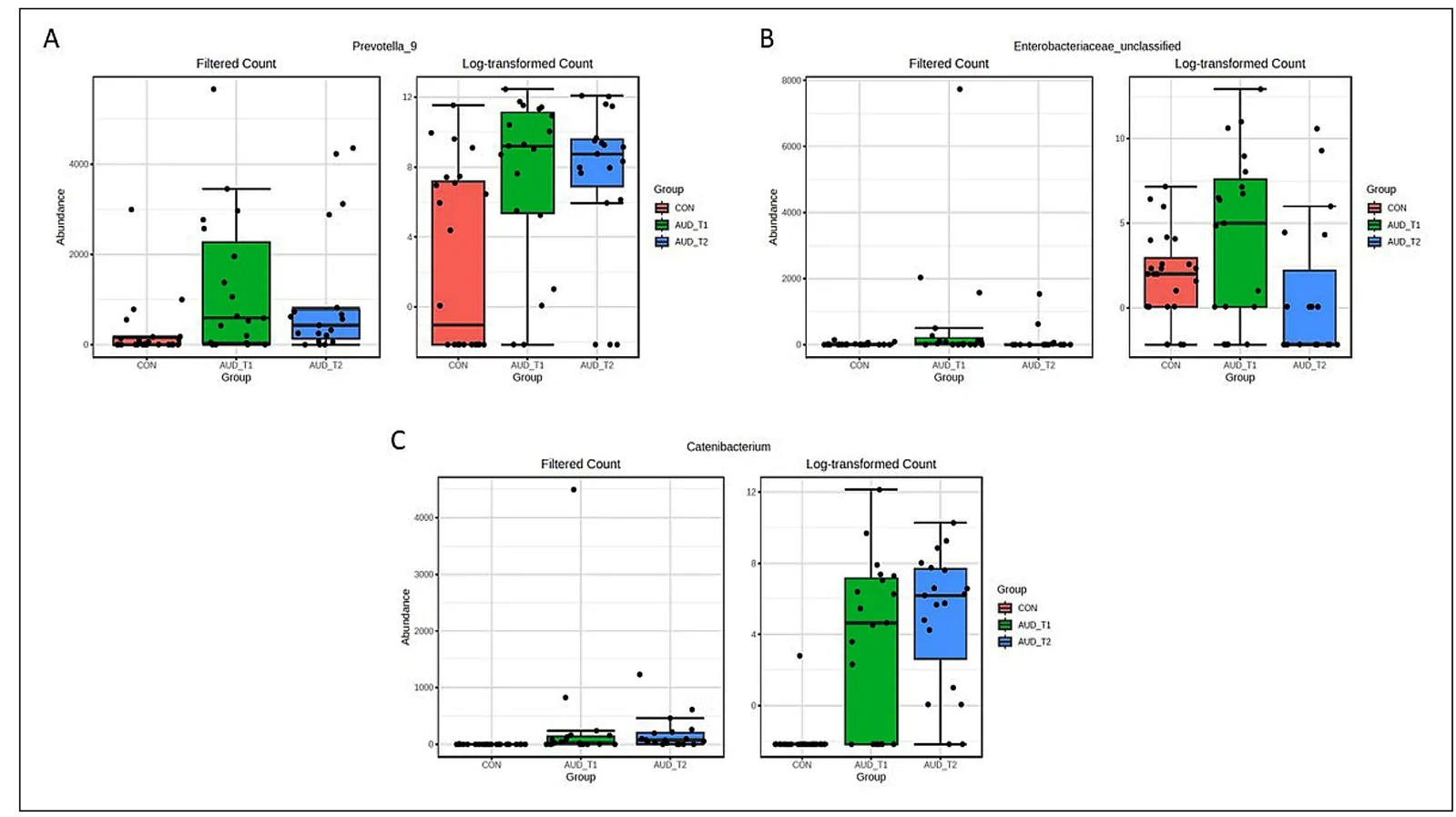
Differential abundant OTUs between the control, AUD-T1, and AUD-T2 groups, by Lefse analysis. (A) genera *Prevotella_9*, (B) genera *Enterobacteriaceae_unclassified*, and (C) genera *Catenibacterium.* Taxa were ranked by LDA score. Differences were considered significant for a logarithmic LDA score threshold of ±2.0 and *P*-value <0.05. AUD-T1: hospital admission for alcohol use disorder treatment. AUD-T2: hospital discharge after alcohol use disorder. CON: control. LDA: linear discriminant analysis. Lefse: linear discriminant effect size. OTUs: operational taxonomic units.

## DISCUSSION

The continuous and excessive consumption of alcohol can lead to damage in various organs and systems^2^ ^,^ ^8^ ^,^ ^11^ ^,^ ^12^. The most significant degree of injury caused by alcohol ingestion is in the liver and gastrointestinal tract, as they are the first to be exposed to high concentrations of this substance^2^ ^,^ ^8^ ^,^ ^17^-^18^. The crucial point is that the abusive use of alcohol disrupts the composition of the intestinal microbiota and the function of the intestinal barrier, which is tightly regulated. This results in the stimulation of bacterial translocation, release of LPS, and endotoxemia, mechanisms that contribute to the development and progression of liver diseases^2^ ^,^ ^3^ ^,^ ^16^. Considering that changes related to the composition of the intestinal microbiota and its metabolism are emerging as one of the most important factors influencing the course of AUD, this study was developed with the aim of evaluating possible alterations in the composition and diversity of the intestinal microbiota at the time of admission and hospital discharge of patients undergoing AUD treatment, in comparison to the healthy control group. Our results show that healthy patients have a significantly higher abundance of *Faecalibacterium*, *Bacteroides*, *Agathobacter*, and *Ruminococcaceae_unclassified* compared to the AUD-T1-group. The opposite was reported for the genera *Prevotella_9*, *Enterobacteriaceae_unclassified*, and *Catenibacterium*. However, in terms of the diversity and abundance of bacterial genera, no significant differences were reported between the time of admission (AUD-T1) and hospital discharge (AUD-T2) for AUD treatment. We observed a significant increase in overall alpha diversity in the healthy control group compared to the AUD-T1 and AUD-T2 groups. This result supports the evidence that chronic alcohol consumption reduces gut microbiota diversity. The modulation of gut microbiota seems to need more time than 14 days to recover from ethanol use, also an activated inflammatory signalling may participate in the microbiota regulation.

Although the relationship between the quantity and duration of alcohol consumption and the development of ALD is clear, it is crucial to note that genetic, epigenetic, and environmental factors also impact its progression^3^ ^,^ ^19^. Approximately 10-20% of individuals with AUD progress to the severe form of ALD^3^ ^,^ ^19^. This data aligns with the findings of this study, as among the population of patients hospitalized for AUD treatment, 11.4% were diagnosed with hepatic cirrhosis. Changes in serum levels of liver enzymes, CRP, and MPO were also observed in patients with AUD. This outcome is expected, as alterations in these serum levels reflect the extent of hepatocellular injury, impaired bile flow, and the presence of low-grade chronic systemic inflammation^17^ ^,^ ^19^ ^,^ ^20^.

In this study, concerning overall alpha diversity, significant differences were observed in the ACE, Shannon, and observed taxa indices among the three analyzed groups. Patients hospitalized for AUD treatment exhibited significantly lower alpha diversity compared to the healthy control group. Data supporting the findings in this study suggest that alcohol consumption leads to both quantitative and qualitative dysbiosis in the intestinal microbiota, as demonstrated in clinical and experimental studies^21^ ^,^ ^24^. The direct interaction of alcohol with the intestinal microbiota and intestinal barrier results in a disturbance in its composition, leading to bacterial translocation, inflammation, and liver damage^2^ ^,^ ^22^ ^-^ ^24^. A recent study aimed at evaluating the composition of the intestinal microbiota in patients with AUD and DHA compared to healthy controls did not find significant differences in alpha bacterial diversity between AUD patients and healthy controls, results that do not align with those obtained in this study^23^. However, Addolorato et al. found similar results to ours, reporting that patients diagnosed with AUD had lower alpha bacterial diversity than healthy control patients^21^.

Recent studies highlight the neuroinflammatory aspect in AUD patients and how the reduced microbial diversity, gut barrier dysfunction, and systemic inflammation, overstimulates neuroinflammation and neurochemical imbalances that perpetuate dependence and relapse^11^. More than that, a recent in vivo study indicates that extracellular vesicles may mediate host-gut microbiota communication associated with alcohol intake^12^. Therefore, chronic alcohol use appears to induce distinct alterations in cell signaling, which in turn influence gut microbiota composition. This may explain the modest differences observed after 14 days of alcohol abstinence in AUD patients, as more pronounced differences are observed when compared with healthy individuals. These systemic signaling alterations are crucial for the re-establishment of intestinal homeostasis. The gut-liver-brain axis describes the mechanisms by which gut dysbiosis promotes neuroinflammation mediated by a persistent low-level systemic inflammation. Our results comparing healthy controls and AUD-T2 suggests that gut dysbiosis due chronic alcohol use constitutes a smoldering inflammatory state that sustains the persistent activation of immune cells and cytokines signaling leading to progressive cellular dysfunction. Despite the differences reported in the literature regarding gut microbiota diversity, it is important to emphasize that distinct populations, due to cultural differences, exhibit different gut microbiota compositions, as dietary habits and climatic and social factors directly influence this composition. Therefore, it is essential to consider the metabolites produced by this intestinal community, since it can promote the metabolism of peptides, neurotransmitters, and short-chain fatty acids that interact with the gut-liver-brain axis^11^. Additionally, in this study, we observed no significant difference in alpha bacterial diversity when comparing the admission and hospital discharge moments for patients undergoing AUD treatment. Studies suggest that a three-week abstinence period following alcohol removal induces a complete recovery of intestinal barrier function in individuals with AUD exhibiting high intestinal permeability^25^. Perhaps this is one of the reasons why we did not find a significant difference between the groups, considering the 14-day hospitalization interval.

The analysis of differential abundance of intestinal microbiota composition conducted in this study allowed for the observation of differences in the abundance of certain bacterial genera between the AUD-T1 group and healthy individuals. In this study, we reported a significant decrease in the abundance of *Faecalibacterium* and *Bacteroides* in patients hospitalized for AUD-T1 treatment compared to healthy individuals, while the opposite was observed for the genera *Prevotella_9*. These findings corroborate with a previous study aimed at evaluating the gut-brain axis in AUD, wherein it was demonstrated that patients with moderate and severe alcohol consumption exhibited intestinal dysbiosis characterized by a decrease in the abundance of *Faecalibacterium* and *Bacteroides* genera and an increase in the abundance of *Prevotella_9*, alterations that influence patient cognitive impairment^26^. Baltazar-Dias et al. identified a beneficial relationship between the increase in the *Faecalibacterium* genera and a detrimental relationship between the increase in the *Prevotella* genera in the intestinal microbiota of patients with alcoholic cirrhosis^27^. The *Faecalibacterium* genera is considered a protective factor against gastrointestinal diseases, and its reduction has been associated with cognitive impairment in users with abusive alcohol consumption^28^. Our study demonstrated a higher abundance of *Faecalibacterium* in healthy individuals compared to those with AUD, indicating that alcohol may destroy bacteria that are considered beneficial to the body, interfering with metabolic pathways that influence disease progression. We reported a higher abundance of *Prevotella_9* in patients hospitalized for AUD treatment. In the literature, the increase in the abundance of bacteria of this genus is associated with inflammation, hypertension, insulin resistance, and the development of liver diseases^29^ ^-^ ^31^. *Prevotella*, despite being immunogenic for producing short-chain fatty acids (SCFAs), is associated with the development of low-grade systemic inflammation in cirrhotic patients^8^ ^,^ ^32^ ^,^ ^33^. Two other SCFAs-producing genera are *Agathobacter* and *Ruminococcaceae*, and in this study, we report a lower abundance of bacteria in AUD patients. The SCFAs are metabolites derived from bacterial fermentation of nondigestible polysaccharides and can influence the pathophysiology of liver disease by acting on maintaining intestinal barrier, immune regulation, anti-inflammatory effects, and regulation of the microbiota itself^34^. In intestinal dysbiosis, oxygen availability in the intestinal lumen increases due to reduced oxidative capacity of colonocytes. Potentially beneficial bacteria, such *as Agathobacter* and *Ruminococcus*, are strict anaerobes, unlike *Enterobacteriaceae*, which are facultative anaerobes^35^ ^,^ ^36^. Therefore, these changes in energy metabolism that favor oxygen in the mucosa may be associated with intestinal dysbiosis in patients who consume alcohol abusively and have alcoholic cirrhosis^27^. Additionally, in this study, we report an increase in the abundance of *Enterobacteriaceae_unclassified* in AUD patients. The relationship between the expansion of bacteria of the *Enterobacteriaceae* family and inflammatory status can be observed using nitrates and nitrites, products of the inflammatory response, which are fundamental in the development of a hyperdynamic circulation, characteristic of decompensation in patients with cirrhosis^37^ ^,^ ^38^.

In this study, we did not report significant differences in bacterial abundance between the AUD-T1 and AUD-T2 groups; only subtle differences were observed. Probably, the 14-day alcohol abstinence period was not sufficient to cause major changes in the composition of the intestinal microbiota, and this can be considered a limitation of the study. In agreement, although not the same parameters, Leclercq et al., interestingly, state that patients with AUD, even after three weeks of abstinence, still have higher levels of ethanol in their feces, suggesting endogenous ethanol production by gut microorganisms^10^. Other limitations of the study are the lack of analysis of inflammatory/endothelial markers on day 14; the use of 16S analysis rather than metagenomics to identify microorganisms and their functions; having only male individuals in the study group, due to hospital admission rules; limited number of participants; the short evaluation period; and the presence of confounding factors such as cirrhosis, diabetes and obesity.

Although there are no significant changes in the composition of the intestinal microbiota, we can observe a trend of dysbiosis recovery, especially if we note the decrease in *Enterobacteriaceae* bacteria abundance in the AUD-T2 group compared to the AUD-T1 group. This decrease in abundance is seen as beneficial, as these bacteria, when in excess, are capable of synthesizing enzymes involved in the production of endotoxins, thereby increasing the production of toxic bacterial metabolites^39^. Other studies also look for an association between alcohol intake and gut microbiota composition and how this can affect different populations, showing that it is a subject of global interest^40^ ^,^ ^41^. Besides the distinct geographical location, the correlation is bonded to cultural and lifestyle factors, for example the kind of beverages that is consumed, if it is obtained from alcoholic fermentation or distillation.

In summary, as demonstrated in clinical and preclinical studies, excessive alcohol consumption or exposure to high levels of alcohol is associated with intestinal dysbiosis, changes in intestinal permeability, and consequent increased endotoxemia^1^ ^,^ ^3^ ^,^ ^23^. This process is associated with the development and progression of various diseases, including ALD. In this study, we observed significant alterations in the diversity and composition of the intestinal microbiota in patients with AUD compared to healthy individuals, along with an increase in inflammatory markers. However, no significant differences were observed in the composition of the intestinal microbiota in AUD patients at the time of hospital admission and discharge, likely due to the short evaluation interval, which was 14 days. Among other study limitations, we highlight the absence of evaluation of inflammatory markers and biochemical variables in patients after hospital discharge, as well as the limited number of participants. Therefore, we suggest conducting further longitudinal studies to assess genetic and epigenetic markers that, although not described in this study, strongly influence the composition and function of the intestinal microbiota. Additionally, we propose evaluating a larger number of patients with established AUD and ALD, considering the gut-liver-brain axis as an integrative approach for a better understanding of the disease’s pathophysiology.

## Data Availability

data-available-upon-request
